# Vasorelaxation Effect of Estrone Derivate EA204 in Rabbit Aorta

**DOI:** 10.1155/2016/7405797

**Published:** 2016-04-14

**Authors:** Juan Li, Wei-Qi Li, Yao Yao

**Affiliations:** ^1^School of Pharmacy, Ningxia Medical University, Yinchuan 750004, China; ^2^Ningxia Engineering and Technology Research Center for Modernization of Hui Medicine, Yinchuan 750004, China; ^3^National Resource Center of Chinese Materia Medica, China Academy of Chinese Medical Sciences, Beijing 100700, China; ^4^China National Center for Biotechnology Development, Beijing 100039, China; ^5^School of Basic Medical Science, Ningxia Medical University, Yinchuan 750004, China; ^6^Research Center of Medical Science and Technology, Ningxia Medical University, Yinchuan 750004, China

## Abstract

Estrogen and its derivatives exert vascular protective effects, but the underlying mechanisms remain to be studied fully.* Objective*. To investigate the vasorelaxation effect and related mechanisms of an estrone derivate EA204[3-(2-piperidin-1-yl)-ethoxy-estra-1, 3, 5 (10)-trien-17-one] on isolated arterial preparation from rabbit thoracic aorta.* Methods*. Aortic rings from rabbit thoracic aorta were prepared and held in small organ bath filled with Krebs solution; tension change was recorded by a multichannel physiological signal collection and handling system.* Results*. EA204 (10^−5^ to 10^−3^ M) induced a concentration-dependent relaxation of aortic rings with endothelium and without endothelium. In denuded arterial preparations, EA204 had a potent relaxing effect on isolated arterial preparations contracted with phenylephrine, norepinephrine, and high-K^+^ solution or BaCl_2_. Mechanism study indicates that EA204 relaxes aortic rings by inhibiting Ca^2+^ channels (both receptor-operating Ca^2+^ channels and the voltage-dependent Ca^2+^ channels were involved) to decrease extracellular Ca^2+^ influx and intracellular Ca^2+^ release. EA204 is different from verapamil, which is a noncompetitive inhibitor of Ca^2+^ channels. In addition, K^+^ channels opening may contribute to this vasorelaxation effect.* Conclusion*. EA204 had a potent endothelium-independent relaxing effect on isolated arterial preparation by inhibiting Ca^2+^ channels and opening K^+^ channels. The results suggest that EA204 is a potential compound for treatment of cardiovascular diseases in postmenopausal women.

## 1. Introduction

It is widely accepted that the low incidence of cardiovascular mortality and morbidity in women can be ascribed to differences in their hormonal status compared to men. Moreover, cessation of ovarian hormones after menopause is known to increase susceptibility to vascular dysfunction such as atherosclerosis [1] and possibly hypertension [2]. Thus, many leading health organizations recommended the use of hormone therapy (HT) for the primary prevention of cardiovascular disorders. Although the results from the Women's Health Initiative (WHI) showed that the health risks (particularly cardiovascular diseases and breast cancer) associated with HT were significantly larger than the health benefits [3], which led to a rapid decrease in HT use worldwide, many scientists have different opinion. For example, a recent long-term follow-up study carried out in Finland revealed that HT could significantly reduce the risk of death caused by coronary heart disease (CHD) or stroke [4], suggesting estrogen as a possible medical source to treat cardiovascular diseases in postmenopausal women.

**Figure 1:**
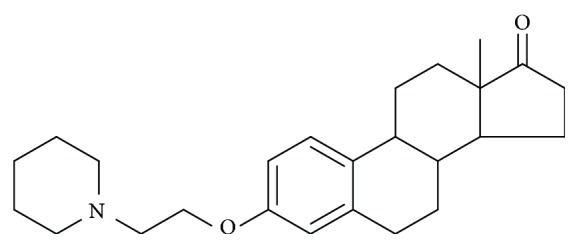
Chemical structure of EA204.

Considering the serious side effects and untoward reactions of natural estrogen, it is reasonable and necessary to synthesize new estrogen derivatives for treatment. Vasorelaxation induced by estrogen and derivatives may be either endothelium-dependent or endothelium-independent [5, 6]. Estrogen receptor has been reported to play a key role in endothelium-dependent vasorelaxation [7, 8]. But the mechanism of endothelium-independent vasorelaxation induced by estrogen remains to be understood fully. For this purpose, we prepared a number of estrone derivatives and tested their relaxing effect. In these 24 estrone derivates EA204[3-(2-piperidin-1-yl)-ethoxy-estra-1, 3, 5 (10)-trien-17-one] (Figure 1) had the strongest relaxing effect. Here we report the relaxing effect of EA204 on isolated arterial preparation from rabbit thoracic aorta and discuss possible mechanism of endothelium-independent vasorelaxation.

## 2. Methods

### 2.1. Animals, Solutions, and Drugs

New Zealand rabbits of both sexes (2.0~2.5 kg) were obtained from the Experimental Animal Center of Ningxia Medical University. All animal experiments in this study were approved by the Ethics Committee of Ningxia Medical University.

A modified Krebs solution with the following ionic composition (mM) was used [9]: NaCl (121.9), KCl (4.7), MgCl_2_ (1.2), CaCl_2_ (2.5), NaHCO_3_ (15.5), KH_2_PO_4_ (1.2), and glucose (11.5). The pH of the solution was adjusted to 7.3–7.4 with 5% CO_2_: 95% O_2_. All solutions were stored at −4°C and fresh dilutions were made daily. These drugs were dissolved in distilled water.

EA204 was provided by Pharmaceutical Engineering College, Shenyang Pharmaceutical University. Phenylephrine, norepinephrine, and acetylcholine were purchased from Sigma-Aldrich Inc. (St. Louis, MO, USA). KCl was from Shenyang Chemical Reagent Factory, China. CaCl_2_ was from Tianjin Bodi Chemical Co., China. BaCl_2_ was from Shenyang Xingdong Reagent Factory, China. Verapamil injection was from Tianjing Heping Pharmaceutical Co., China. Glibenclamide was from Beijing Taiyang Pharmaceutical Co., China. Sodium pentobarbital was from Beijing Chemical Reagents Co., China.

### 2.2. Preparations of the Aorta Rings and Assessment of Vascular Function

Rabbits were killed by overdose of sodium pentobarbital (25 mg/kg) injected via the ear vein. The thoracic aortas were dissected and isolated by removing the surrounding connective tissue. They were cut into rings (3 mm) and were mounted between two stainless-steel triangles in a small organ bath containing 10 mL Krebs solution (37°C), aerated with 95% O_2_ and 5% CO_2_, one of which was fixed to the organ bath and the other to a force displacement transducer (Chengdu Instrument Plant). Isometric tension change was recorded by RM6240B Multichannel Physiological Signal Collection and Handling System (Chengdu Instrument Plant). Each preparation was stretched to an initial tension of 1 g [10] and allowed to equilibrate in warmed Krebs solution (37°C) for 1 h.

The effects of EA204 on the rings with intact or denuded endothelium have been examined. In order to test the endothelium-independent vasorelaxation induced by EA-204, endothelium was mechanically removed by rubbing with a steel wire. Aortic rings were precontracted with phenylephrine (1 *μ*M) and then acetylcholine (20 *μ*M) was added into the organ bath to determine whether the aorta was intact or denuded. If the maximal relaxant effect was more than 80% of initial contraction, we considered functional endothelium to be present. Failure of arteries to relax to acetylcholine was considered to indicate denuded aorta [11]. Then the concentration-response curves of EA204 were obtained as the following protocol: (1) following 1 h equilibration, the aortic preparations were precontracted with phenylephrine (1 *μ*M); (2) after the contraction had reached a stable plateau, the strips were washed by Krebs solution and 30 min later the strips were precontracted again; (3) when a stable contraction was obtained, the cumulative concentrations of EA204 (10^−5^ to 10^−3^ M) were added and relaxation produced by each concentration of EA204 was measured and expressed as a percentage of the maximum possible relaxation (i.e., relaxation back to the baseline tension). In other three groups, the experiments were repeated with norepinephrine (3 *μ*M), high-K^+^ (60 mM), and BaCl_2_ (2 mg/mL), respectively.

To investigate how EA204 affects Ca^2+^ channels, verapamil, a noncompetitive calcium antagonist, was used to compare with EA204. After 1 h equilibration, the aortic preparations were incubated in a Ca^2+^-free Krebs solution for 30 min and then in a high-K^+^ and Ca^2+^-free Krebs solution for 20 min. After that, CaCl_2_ was added in a cumulative fashion (10^−6^ to 10^−2^ M) to obtain control concentration-response curves of Ca^2+^. Then, rings were washed by Krebs solution and equilibrated for 1 h. Following the incubation of EA204 (10^−5^ M) for 20 min, the concentration-response curves of Ca^2+^ were repeated. Verapamil (10^−7^ M) was used to compare with EA204 as a noncompetitive inhibitor of Ca^2+^ channels.

To determine whether K^+^ channels are involved, the affection of glibenclamide on the vasorelaxation effect of EA204 was investigated. After 1 h equilibration, the isolated preparations were precontracted with BaCl_2_ (2 mg/mL) or KCl (60 mM). After the contraction had reached a stable plateau, EA204 was added in a cumulative fashion (10^−5^ to 10^−3^ M) to obtain control concentration-response curves of EA204. Then, the isolated preparations were washed by Krebs solution and equilibrated for 1 h. Following the incubation of glibenclamide (10^−5^ M) for 10 min, the concentration-response curves of EA204 were repeated [12].

### 2.3. Statistical Analysis

The data are presented as the means ± SEM for the number of experiments indicated; *n* refers to the number of preparations from different animals used.

## 3. Results

**Figure 2:**
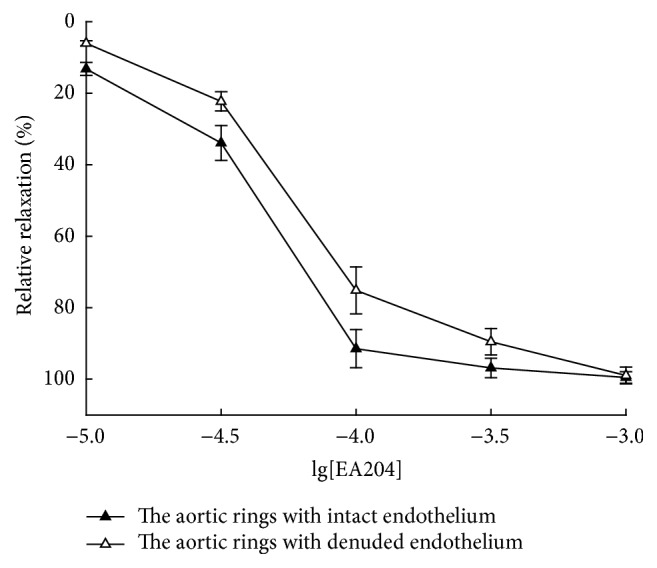
Concentration-response curves for EA204 in rabbit aorta with and without endothelium. Aortic rings were precontracted with phenylephrine (1 *μ*M). Endothelium was removed mechanically by rubbing with a steel wire. Responses are expressed as a percentage of the maximum possible relaxation, that is, relaxation back to the baseline tension. Values indicated the means ± SEM (*n* = 8).

The effects of EA204 on the rings with intact or denuded endothelium were examined. Results have shown that EA204 (10^−5^ to 10^−3^ M) induced a concentration-dependent relaxation of rings with endothelium and without endothelium (as shown in Figure 2). Low concentration EA204 (10^−5^ to 3 × 10^−4^ M) induced a weaker relaxation in denuded aorta, but the maximal responses were identical under high concentration (10^−3^ M) condition.

**Figure 3:**
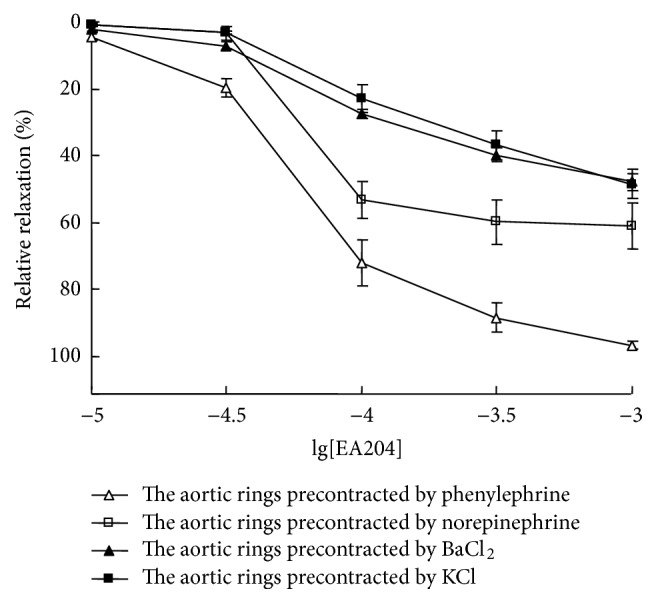
Endothelium-independent vasorelaxant effects of EA204 on denuded aortic rings of rabbits precontracted by phenylephrine (1 *μ*M), norepinephrine (3 *μ*M), high-K^+^ solution (60 mM), and BaCl_2_ (2 mg/mL), respectively. Endothelium was removed mechanically by rubbing with a steel wire. Responses are expressed as a percentage of the maximum possible relaxation, that is, relaxation back to the baseline tension. Values indicated the means ± SEM (*n* = 8).

To investigate endothelium-independent vasorelaxation effect of EA204, denuded aortic rings from rabbit thoracic aorta were prepared and tension changes were recorded. The results indicated that administration of EA204 induced relaxation of the isolated aorta preparations precontracted with phenylephrine, norepinephrine, high-K^+^, or BaCl_2_, and the maximum relaxant effect was found in rings precontracted with phenylephrine (as shown in Figure 3).

**Figure 4:**
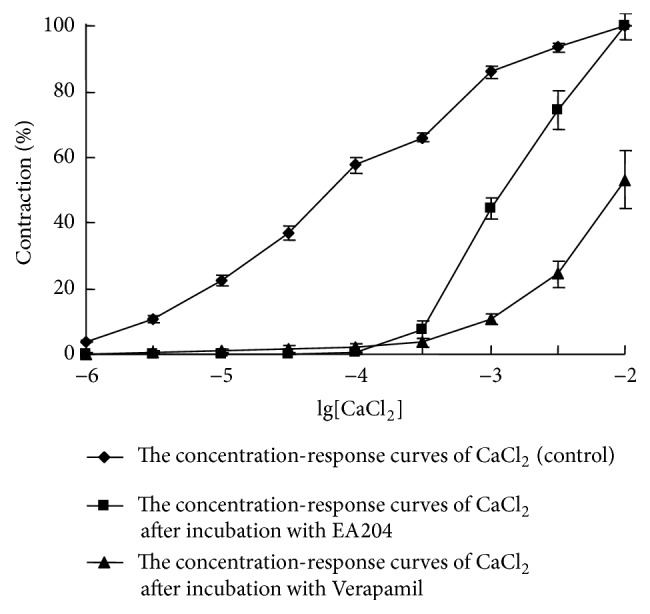
Inhibitory activities of EA204 (10 *μ*M) and verapamil (0.1 *μ*M) on the concentration-response curves of CaCl_2_ (10^−6^ to 10^−2^ M) on denuded aortic rings of rabbits. Endothelium was removed mechanically by rubbing with a steel wire. Responses are expressed as a percentage of the maximum contraction induced by CaCl_2_ (10^−2^ M). Values indicated the means ± SEM (*n* = 8).

To investigate whether EA204 affects Ca^2+^ channels, we did experiments to compare EA204 with verapamil, which is a noncompetitive calcium antagonist. Increasing concentration of CaCl_2_ (10^−6^ to 10^−2^ M) resulted in concentration-dependent contraction of the isolated aorta of rabbits. Both EA204 and verapamil could shift the concentration-response curves of CaCl_2_ to the right, and the rightward shifts were concentration-dependent. It suggested that EA204 and verapamil inhibited voltage-dependent Ca^2+^ channels and decreased Ca^2+^ influx. However, after incubation with verapamil the maximum responses of the cumulative concentration-response curves were significantly decreased, while EA204 did not affect the maximum responses (as shown in Figure 4). The results indicate that, different from verapamil, EA204 is not a noncompetitive calcium antagonist.

**Figure 5:**
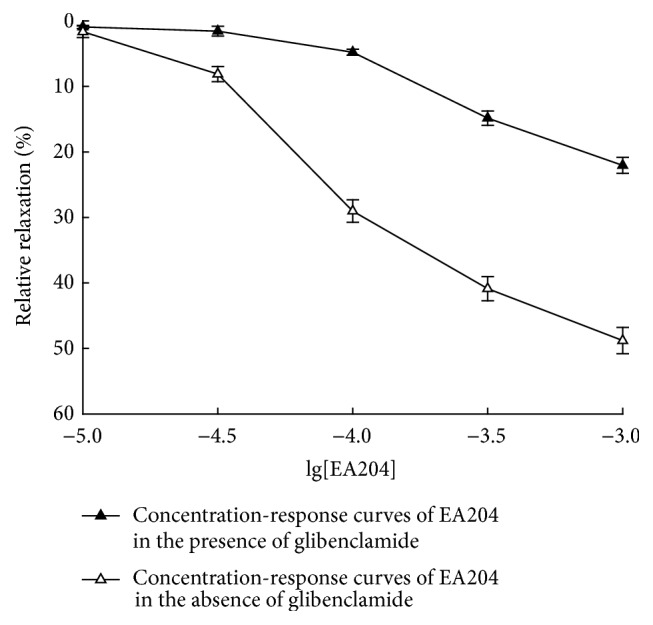
Cumulative concentration-response curves of EA204 in the absence or presence of glibenclamide (10^−5^ M) on denuded aortic rings of rabbits precontracted with BaCl_2_ (2 mg/mL). Endothelium was removed mechanically by rubbing with a steel wire. Responses are expressed as a percentage of the maximum possible relaxation, that is, relaxation back to the baseline tension. Values indicated the means ± SEM (*n* = 8).

**Figure 6:**
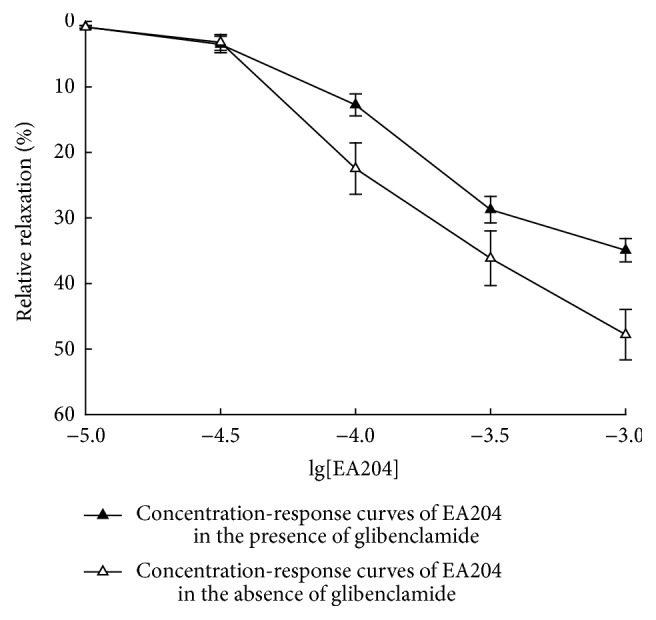
Cumulative concentration-response curves of EA204 in the absence or presence of glibenclamide (10^−5^ M) on denuded aortic rings of rabbits precontracted with KCl (60 mM). Endothelium was removed mechanically by rubbing with a steel wire. Responses are expressed as a percentage of the maximum possible relaxation, that is, relaxation back to the baseline tension. Values indicated the means ± SEM (*n* = 8).

To determine whether K^+^ channels are involved, we investigated the affection of glibenclamide on the vasorelaxation effect of EA204. Glibenclamide is a K^+^ channels antagonist which could selectively block ATP sensitive K^+^ channels to increase extracellular Ca^2+^ influx. It was found that the vasorelaxation effect of EA204 was significantly decreased after the incubation of glibenclamide (as shown in Figures 5 and 6). The results indicate that K^+^ channels opening may contribute to the vasorelaxation effect of EA204.

## 4. Discussion

The cardiovascular protective activity of estrogen is reported to be mediated by an effect on the vessel wall itself directly and an indirect effect on the lipoprotein metabolism. Recently, researchers have made numerous efforts to understand estrogen's cardiovascular actions, especially in its endothelial mechanisms [13, 14]. For example, 17*β*-estradiol has been shown to augment endothelium-dependent vasodilation [15], promote endothelial integrity [16, 17], and attenuate the inflammatory response in endothelium [18]. However, the endothelium-independent mechanisms are far from being understood.

In smooth muscle cells, there are two major kinds of Ca^2+^ channels: the voltage-dependent Ca^2+^ channels and receptor-operated Ca^2+^ channels. Phenylephrine and norepinephrine cause vasoconstriction by opening receptor-operated calcium channels [19, 20]. High-K^+^ solution can excite voltage-dependent Ca^2+^ channels and increase extracellular Ca^2+^ influx; BaCl_2_ causes the depolarization of the cell membrane and intracellular Ca^2+^ release; moreover, BaCl_2_ can transit the cell membrane through the Ca^2+^ channels to bind with troponin directly. Researchers have demonstrated that the relaxant effect of 17*β*-estradiol on human saphenous vein was elicited by calcium-dependent pathways [21]. Consistent with previous study, our results suggested that EA204 had a potent relaxation effect in endothelium-independent way, and this effect may be associated with the Ca^2+^ channels.

In order to know more about the relationship between relaxation effect of EA204 and Ca^2+^ channels, a noncompetitive calcium antagonist verapamil was used to compare with EA204. CaCl_2_ was added in a cumulative fashion to obtain control concentration-response curves of Ca^2+^. As reported before, increasing concentration of CaCl_2_ resulted in concentration-dependent contraction of the isolated aorta [19]. Moreover, both EA204 and verapamil shifted the concentration-response curves of CaCl_2_ to the right, and the effects were dose dependent. The results suggested that EA204 and verapamil inhibited voltage-dependent Ca^2+^ channels and decreased Ca^2+^ influx. However, after incubation with verapamil, the maximum responses of the cumulative concentration-response curves to CaCl_2_ were significantly decreased, which were not affected after incubation with EA204. The result indicates that, different from verapamil, EA204 is not a noncompetitive calcium antagonist.

Glibenclamide is a K^+^ channels antagonist, which can selectively block ATP sensitive K^+^ channels to increase extracellular Ca^2+^ influx [22]. K^+^ channels are usually involved in the relaxing effect; thus we investigated the effect of glibenclamide on EA204 induced vasorelaxation to discuss if K^+^ channels are responsible for this effect. We found that the vasorelaxation effect of EA204 was significantly decreased after the incubation of glibenclamide. The results indicate that K^+^ channels opening is involved in the mechanisms of vasorelaxation effect.

Taken together, the endothelium-independent vasorelaxant action and underlying mechanism of estrone derivate EA204 in rabbit aorta were elucidated for the first time in this study. The mechanism includes the inhibition of Ca^2+^ influx through voltage-dependent or receptor-operated Ca^2+^ channels, the inhibition of intracellular Ca^2+^ release, and the opening of K^+^ channels. These data suggest that EA204 is a potential compound to be used for the treatment of cardiovascular diseases in postmenopausal women.
